# A landscape on disorders following different COVID-19 vaccination: a systematic review of Iranian case reports

**DOI:** 10.1186/s40001-023-01531-7

**Published:** 2023-11-26

**Authors:** Mona Sadat Larijani, Delaram Doroud, Mohammad Banifazl, Afsaneh Karami, Anahita Bavand, Fatemeh Ashrafian, Amitis Ramezani

**Affiliations:** 1 Clinical Research Department, Pasteur Institute of Iran, No: 69, Pasteur Ave, Tehran, 1316943551 Iran; 2 Quality Control Department, Production and Research Complex, Pasteur Institute of Iran, Tehran, Iran; 3 Iranian Society for Support of Patients With Infectious Disease, Tehran, Iran; 4 Department of Infectious Disease, Zanjan University of Medical Sciences, Zanjan, Iran

**Keywords:** Adverse event, SARS-CoV-2, Organ involvement, Vaccine monitoring

## Abstract

There have been massive studies to develop an effective vaccine against SARS-CoV-2 which fortunately led to manage the recent pandemic, COVID-19. According to the quite rapidly developed vaccines in a fast window time, large investigations to assess the probable vaccine-related adverse events are crucially required. COVID-19 vaccines are available of different platforms and the primary clinical trials results presented acceptable safety profile of the approved vaccines. Nevertheless, the long-term assessment of the adverse events or rare conditions need to be investigated. The present systematic review, aimed at classification of probable vaccine-related unsolicited adverse events in Iranian population through the data collection of the published case report studies.

The related published case reports were explored via PubMed, Web of Science and Google scholar according to the available published data up to 14^th^ Dec, 2022 using PRISMA guideline. Out of 437 explored studies, the relevant data were fully investigated which totally led to 40 studies, including 64 case reports with a new onset of a problem post-vaccination. The cases were then classified according to the various items, such as the type of adverse event and COVID-19 vaccines.

The reported COVID-19 vaccines in the studied cases included BBIBP-CorV, ChAdOx1-S, Sputnik V and COVAXIN. The results showed that the adverse events presented in 8 different categories, including cutaneous involvements in 43.7% (*n* = 28), neurologic problems (*n* = 16), blood/vessel involvement (*n* = 6), cardiovascular involvement (*n* = 5), ocular disorders (*n* = 4), liver disorder/failure (*n* = 2), graft rejection (*n* = 2) and one metabolic disorder. Notably, almost 60% of the cases had no comorbidities. Moreover, the obtained data revealed nearly half of the incidences occurred after the first dose of injection and the median duration of improvement after the symptom was 10 days (range: 2–120). In addition, 73% of all the cases were either significantly improved or fully recovered. Liver failure following ChAdOx1-S vaccination was the most serious vaccine adverse event which led to death in two individuals with no related medical history.

Although the advantages of COVID-19 vaccination is undoubtedly significant, individuals including with a history of serious disease, comorbidities and immunodeficiency conditions should be vaccinated with the utmost caution. This study provides a comprehensive overview and clinical implications of possible vaccine-related adverse events which should be considered in further vaccination strategies. Nevertheless, there might be a bias regarding potential under-reporting and missing data of the case reports included in the present study. Although the reported data are not proven to be the direct vaccination outcomes and could be a possible immune response over stimulation, the people the population with a medium/high risk should be monitored after getting vaccinated against COVID-19 of any platforms. This could be achieved by a carefull attention to the subjects ‘ medical history and also through consulting with healthcare providers before vaccination.

## Background

COVID-19 as the most recent global pandemic, typically presents as lower respiratory tract infection which may lead to severe symptoms [1, 2]. To date, vaccines have been one of the most effective ways to control the infectious diseases [3, 4]. Fortunately, vaccination against COVID-19 was explored at the right time and led to fast outcomes through different platforms and hopefully pandemic control [5, 6]. Nevertheless, booster shots are still recommended as the immunity wanes over the time and new variants are capable to escape from immune system [7, 8].

From another point of view, the quick procedure of vaccine development could possibly have lately unsolicited events beside the immunity protection. Many studies have shown SARS-CoV-2 manifestations through which the virus affects the host in various presentations even in a late episode [9, 10]. As the number of vaccinated individuals grows up, the knowledge of possible and probable vaccine effect develops through case reports and safety studies [11, 12].

Although the exact mechanism through which the vaccine components can manipulate human body is not clear yet, the cumulative and comparative data would bring sufficient data especially by the follow-up programs.

Early studies on COVID-19 vaccines-related adverse events (AEs) mainly reported local reaction at the site of the injection and some temporary systemic side effects which normally lasted few days, among which fever, headache and fatigue were the most common ones. Moreover, further investigation indicated that the AEs are mostly mild, hence individual daily activities are not normally interfered with [13, 14]. A cross-sectional study in Nepal, presented higher rate of vaccine adverse events after the first dose of both vaccines, while a follow-up study from Iran demonstrated that the vast majority of the vaccine-related AEs were set after receiving the booster shots [15, 16]. It should be also noticed that, COVID-19 vaccination has been the most recently administrated vaccine worldwide and the massive studies and reports on the related side effects are naturally highlighted. However, comparison of advantages and disadvantages of COVID-19 vaccines has shown that it is still recommended. It has been assumed that there will be more in cardiovascular diseases due to spike proteins encoded in vaccines [17, 18]. Furthermore, there is a possible threat of unknown organ hurt caused by the immunization which is still hidden. Thus, any type of study in this era regarding vaccine safety seems highly practical for future vaccine programs. Along with the different type of the conducted studies on COVID-19 vaccination, case report studies have been massively published. Owing to the fact that this kind of study provides a detailed report of many aspects, including symptoms, diagnosis and follow-up of individuals, they could possibly bring a new insight to the COVID-19 vaccines-related side effects. Furthermore, these reports usually describe a novel or unusual incidences in a faster window time than cross-sectional research or follow-up studies. According to several case reports post-vaccination against COVID-19, the present study aimed to classify the new onset of disorders in Iranian individuals with no previous related medical history. The present data provides a better overview of documented COVID-19 vaccine-related disorders along with the cases’ characteristics and the treatment/follow-up after administration of primary and/or booster doses. Furthermore, the screened disorders post vaccination are classified based on the organ involvement to facilitate the recognition of adverse events prevalence, time of the incidence and the final outcome.

## Methods

### Search strategy

The present study was conducted according to preferred reporting items for systematic reviews and meta*-*analyses (PRISMA) in all relevant items [19].

Three databases, PubMed, Web of Science and Google scholar, were explored. The initial search started in December, 2022 and all the available data up to December 14, 2022 were collected. The relevant data were targeted with terms of: “COVID-19 vaccine”, “SARS-CoV-2”, “case reports”, “adverse events” and “Iran”. In order not to miss any published relevant data we explored each searching item solely and also in combination forms.

### Data collection

**Fig. 1:**
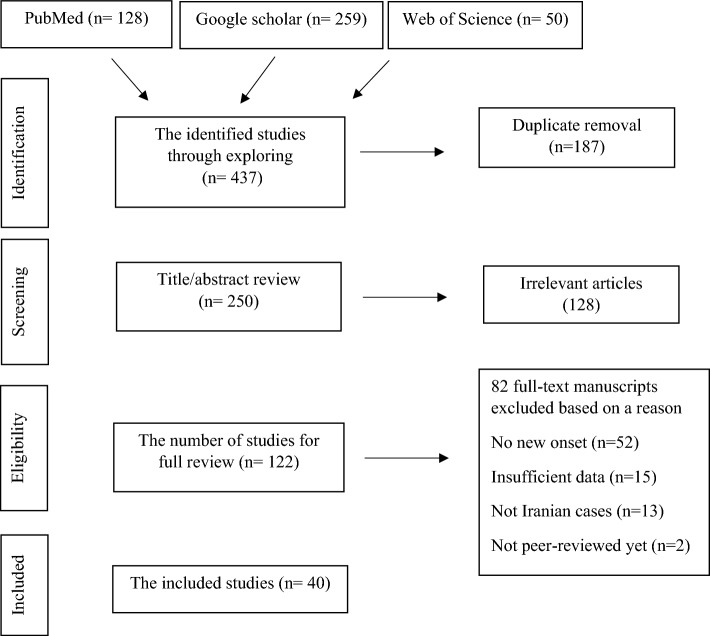
The review flowchart based on PRISMA

To exclude the irrelevant data, titles and abstracts were initially screened. To maximize the validity, the preprints or unpublished data were not included. At the next step, the full texts of the articles were evaluated regarding the eligibility of inclusion in the study. The full text screening and data extraction was done by the end of the February, 2023. To collect the relevant studies two main principles were considered in inclusion criteria. First, the reported adverse events only were considered post-vaccination against COVID-19, not the infection itself. Thus, data including the case reports after COVID-19 disease were also removed. Moreover, only the new onsets were considered which means the cases who had a history of the exactly same disorder were not included (Fig. 1).

The following data were extracted according to a valid datasheet, including: age, gender, vaccine type, date of injection, date of disorder appearance, duration of the symptoms, type of the developed disorder, medical history of the case, hospitalization, response to the treatment, follow-up and outcomes. All the mentioned items were exactly collected from the case report studies and were re-checked thoroughly and was completed by March 2023.

### Data analysis

Statistical analyses were performed using SPSS software (version 22). Descriptive analyses are presented by numbers, percentages, frequencies and mean (SD) or median (min‐max) were applied to report the quantitative variables.

## Results

The initial search yielded 437 studies. After duplication removal, 250 papers were investigated regarding the titles and abstracts from which 128 articles were excluded according to the eligibility of the criteria including the case reports which had the same medical history or those with insufficient data. Eventually 40 manuscripts met the criteria of the systematic review.

A total of 64 cases including 31 females and 33 males with a mean age of 47.67 ± 17.69 and a range of 18 to 91 were investigated from whom 60% had no remarkable medical history. The previous history of COVID-19 was rare among the cases and all the COVID-19 PCR tests were negative at the time of manifestation (Tables 1, 2, 3 and 4).

**Table 1:** Cutaneous manifestations following COVID-19 vaccines in Iran

Case no	Type of disorder	Age	Gender	Comorbidity	COVID-19 test/history	Vaccine type	Time of incidence	Ref.
1	Extensive rash and edema	77	Female	Hypertension	Negative	ChAdOx1-S	2 days after the 1st dose	[20]
2	Radiation Recall Dermatitis	50	Female	History of breast cancer and radical mastectomy	Not stated	BBIBP-CorV	1 week after the 2nd dose	[21]
3	Erythemato-violaceous and sclerotic lesions	70	Female	-	Negative	ChAdOx1-S	2 days after the 1st dose	[22]
4	Panniculitis	40	Female	-	Not stated	Sputnik V	13 days after the 1st dose	[23]
5	Alopecia areata	23	Female	-	Not stated	ChAdOx1-S	1 week after the 1st dose	[24]
6		74	Male	Fatty liver	Not stated	BBIBP–CorV	2 days after the 2nd dose	[25]
7		37	Male	-	Not stated	BBIBP–CorV	6 days after the both doses	[25]
8	Herpes simplex	63	Female	Rheumatoid arthritis	Not stated	BBIBP–CorV	7 days after the 2nd dose	[26]
9	Toxic Epidermal Necrolysis (TEN)	76	Male	Atorvastatin 10 mg/day taken for several years	Not stated	BBIBP–CorV	1 day after vaccination	[27]
10		71	Male	–	Not stated	BBIBP–CorV	10 days after the 1st dose	[25]
11	Pemphigus vulgaris (PV)	76	Female	Diabetes mellitus, hyperlipidemia, and ischemic heart disease	Not stated	BBIBP–CorV	1 month after the 2nd dose	[28]
12		30	Female	–	Not stated	BBIBP–CorV	16 days after 1st dose	[25]
13	New-onset lichen planus (LP)	52	Female	–	Positive	BBIBP–CorV	1 week after the 2nd dose	[29]
14		45	Female	Hypertension	Not stated	BBIBP–CorV	14 days after the 1st dose	[25]
15		40	Male	–	Not stated	BBIBP–CorV	10 days after the both	[25]
16		45	Male	–	Not stated	BBIBP–CorV	7 days after the both	[25]
17		45	Male	–	Not stated	ChAdOx1-S	7 days after the 1st dose	[25]
18		49	Female	–	Not stated	BBIBP–CorV	10 days after the 1st dose	[25]
19	Psoriasis exacerbation	50	Male	Arthritis	Not stated	BBIBP–CorV	4 days after the first dose, 6 days after the 2nd dose	[25]
20	Bullous pemphigoid	85	Female	–	Not stated	BBIBP–CorV	20 days after the 1st dose	[25]
21		91	Male	–	Not stated	BBIBP–CorV	19 days after the 1st dose	[25]
22	Cutaneous vasculitis	45	Male	–	Not stated	BBIBP–CorV	2 days after the 1st dose	[25]
23	Pityriasis rosea	26	Male	Hypertension, diabetes mellitus	Not stated	BBIBP–CorV	14 days after the booster	[25]
24	Herpes zoster	60	Female	–	Not stated	BBIBP–CorV	6 days after the 1st dose	[25]
25	Urticaria and erythema multiform	31	Male	–	Not stated	BBIBP–CorV	11 days after the 2nd dose	[25]
26		32	Female	–	Not stated	ChAdOx1-S	20 days after the 1st	[25]
27	Morphea	35	Female	Hyperlipidemia, diabetes	Not stated	ChAdOx1-S	10 days after the 1st	[25]
28	Steven–Johnson syndrome	63	Female	Mild plaque-type psoriasis type II diabetes mellitus	Not stated	BBIBP–CorV	24h after vaccination	[30]

**Table 2:** Neurological disorders following COVID-19 vaccines in Iran

Case no	Type of disorder	Age	Gender	Comorbidity	COVID-19 test/history	Vaccine type	Time of incidence	Ref.
1	Facial Paresis	34	Female	Migraine attacks (under treatment)	Not stated	Sputnik V	1 day after the 1st dose	[31]
2	Encephalopathy	27	male	–	Not stated	ChAdOx1-S	8 days after the 1st dose	[32]
3		56	Female	–	Negative	ChAdOx1-S	2 days after the 1st dose	[20]
4	Transverse myelitis	31	Female	–	Negative	ChAdOx1-S	3 weeks after the 1st dose	[33]
5	Acute vestibular neuritis	51	Male	–	Negative	ChAdOx1-S	11 days after the 1st dose	[34]
6	Bell's palsy	27	Female	–	Negative	Sputnik V	3–5 days after the 1st dose	[35]
7		58	Male	Controlled diabetes mellitus	Not stated	Sputnik V	10 days after the 1st dose	[35]
8	Thalamic hemi-chorea	72	Male	History of laparoscopic cholecystectomy	Negative	ChAdOx1-S	9 days after the 1st dose	[36]
9	Guillain–Barre syndrome	60	Male	Controlled hypertension and hypothyroidism	Negative	BBIBP–CorV	20 days after the booster	[37]
10		46	Male	–	Negative	ChAdOx1-S	3 days after the 2nd dose	[38]
11		36	Male	–	Negative	BBIBP–CorV	5 days after the 1st dose	[38]
12		32	Male	–	Negative	BBIBP–CorV	14 days after the 1st dose	[38]
13		68	female	–	Negative	ChAdOx1-S	4 days post the 2nd	[39]
14	Aseptic meningitis	26	Female	–	Negative	ChAdOx1-S	A few hours the 1st dose	[40]
15	Extensive myelitis	71	Male	Diabetes mellitus, hypertension and Ischemic Heart Disease	Not stated	BBIBP–CorV	5 days after the 1st dose	[41]
16	Acute disseminated encephalomyelitis	37	Male	–	Negative	BBIBP–CorV	few days to 1 month after the 1st dose	[42]

**Table 3:** Vessels/cardiac disorders following COVID-19 vaccines in Iran Blood involvement

Case no	Type of disorder	Age	Gender	Comorbidity	COVID-19 test/history	Vaccine type	Time of incidence	Ref.
1	Thrombotic thrombocytopenia	70	Female	Diabetes mellitus type 2, hypertension, and coronary artery disease	Not stated	ChAdOx1-S	1 day after the 1st dose	[20]
2	Vasculitis	55	Female	controlled sarcoidosis	Not stated	BBIBP–CorV	3 days after the 1st dose	[43]
3	Cerebral venous sinus thrombosis	55	Female	Hypertension/a surgery history of hysterectomy 10 years ago	Negative	ChAdOx1-S	After the 1st dose	[44]
4	Acquired thrombotic thrombocytopenic purpura (aTTP)	22	Female	–	Negative	ChAdOx1-S	3 weeks after the 1^st^ dose	[45]
5	Purpuric dermatosis &lymphocytic vasculopathy	53	Female	History of treated breast cancer	Not stated	BBIBP–CorV	9 days after the 1st dose	[46]
6		50	Male	–	Not stated	BBIBP–CorV	2 months after vaccination	[46]
7	Myocarditis	29	Male	–	Negative	Sputnik V	2 days after the 2nd dose	[47]
8		26	Male	–	Negative	ChAdOx1-S	4 days after the 2nd dose	[48]
9		32	Female	–	Negative	ChAdOx1-S	3 days after the 1st dose	[49]
10	Atrioventricular block	65	Male	–	Not stated	BBIBP–CorV	A few days after vaccination	[50]
11	Long QT interval and syncope	70	Male	Hypertension (HTN) and diabetes mellitus under medical treatment	Negative	ChAdOx1-S	3 days after the 1st	[51]

**Table 4:** Other complications following COVID-19 vaccines in Iran involvement

Case no	Type of disorder	Age	Gender	Comorbidity	COVID-19 test/history	Vaccine type	Time of incidence	Ref
Ocular involvement								
1	Paracentral acute middle maculopathy	38	Male	–	Negative	BBIBP–CorV	2 weeks after vaccination	[54]
2	Herpetic endotheliitis and stromal keratitis	30	Female	Hypothyroidism	Not stated	BBIBP–CorV	2 weeks after vaccination	[55]
3	Intracranial hypertension and papilledema	32	Male	–	Not stated	Sputnik V	3 days after the 1st dose	[56]
4	Acute macular neuroretinopathy	18	Female	–	Negative	BBIBP–CorV	5 days after the 1st dose	[57]
Liver involvement								
5	Fulminant hepatitis	35	Male	Controlled psychological problems	Not stated	ChAdOx1-S	8 days after the 1st dose	[53]
6	Acute liver failure	34	Male	–	Not stated	ChAdOx1-S	2 days after the 1st dose	[52]
Thyroid disorder								
7	Subacute thyroiditis	34	Female	–	Negative	COVAXIN	11 days after the 1st dose	[58]
Graft rejection								
8	Corneal Graft Rejection	36	Female	Penetrating keratoplasty (PKP) secondary to herpes simplex keratitis (HSK)	Not stated	BBIBP–CorV	7 days after the 1st dose	[59]
9		54	Female		Not stated	BBIBP–CorV		

The reported COVID-19 vaccines in the studied cases included BBIBP–CorV (Sinopharm).

(*n* = 35), ChAdOx1 nCoV-19 (AstraZeneca) (*n* = 22), Sputnik V (*n* = 6) and COVAXIN (*n* = 1).

The median of duration between the vaccination and any appeared event was 7 days (range: 1–60). Of 64 cases, 52 ones experienced a type of manifestation post-first dose, 10 post-second dose and only 2 after the booster shot.

COVID-19 vaccine triggered different manifestations from which cutaneous disorders (Table 1) were spotted as the most frequent one accounting for 43.7% (*n* = 28) followed by neurologic problems (Table 2) in 25% of the cases (*n* = 16). Other unsolicited events included blood/vessel involvement (*n* = 6), cardiovascular involvement (*n* = 5), ocular disorders (*n* = 4), liver disorder/failure (*n* = 2), graft rejection (*n* = 2) and one metabolic disorder (Tables 3 and 4). The median duration of improvement after the symptom onset was 10 (range: 2–120) days.

**Fig. 2:**
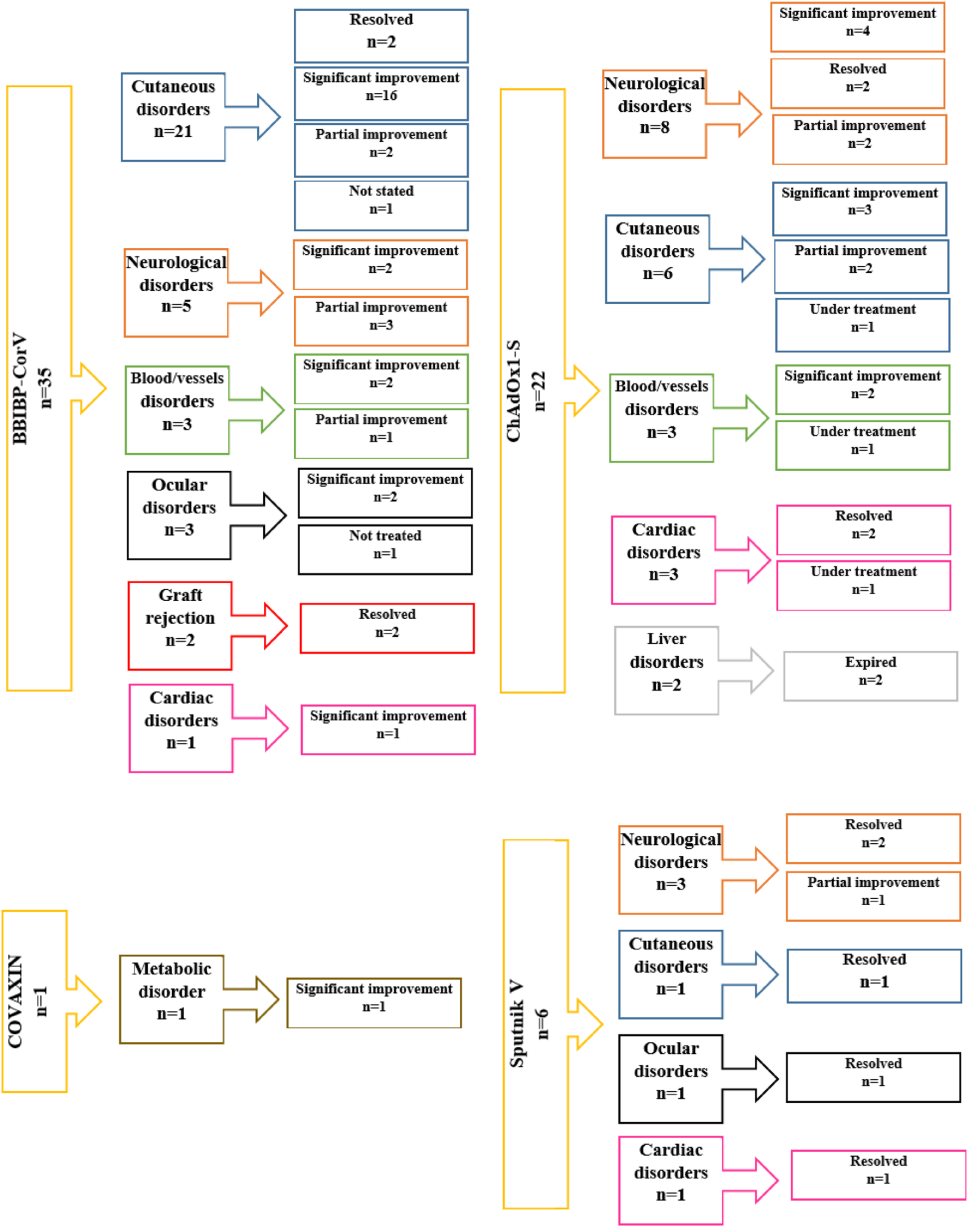
COVID-19 vaccines and their potential association with disorders in case reports. Each complication is shown with a different color to simplify comparison between the vaccines. The final reported status for each disorder is also presented

Cutaneous involvement presented in various forms, such as alopecia, lichen planus, rash, dermatitis and stromal keratitis. Notably, the dermal manifestation occurred equally on both men and women among whom only one person had a history of COVID-19. The other interesting finding is that rare diseases were also screened such as Steven–Johnson syndrome, Morphea and Toxic Epidermal Necrolysis (TEN). BBIBP–CorV, ChAdOx1-S and Sputnik V vaccines led to 21, 6 and 1 cutaneous disorders, respectively. Finally, almost 90% of the skin manifestations were fully or significantly improved after the applied treatment (Fig. 2, Table 1).

In addition to type of disorders, we also evaluated the recovery time as well. To achieve that, the provided data were categorized to 6 outcomes as resolved, significant improvement, partial improvement, under treatment, not-treated and expired. Based on the outcome statement of the studied cases, of 64 incidences, 13 were resolved, 33 were significantly improved and 10 were partially improved. 4 cases were under treatment, one remained untreated and 2 cases expired. Three studies did not mention the outcome. Therefore, 73% of all the cases were either significantly improved or fully recovered from the incidence.

According to the available statements, 20 cases were hospitalized and 22 ones were recommended to be followed-up in the schedule varying from 14 days to 6 months.

Neurological problems were mostly induced by ChAdOx1-S (*n* = 8) followed by BBIBP–CorV (*n* = 5) and Sputnik V (*n* = 3). Guillain–Barre syndrome and Bell’s palsy were the most common ones. Totally, 62.5% of these problems met significant improvement or were resolved (Fig. 2, Table 2).

The most serious vaccine outcomes might be acute liver failure which was captured in two ChAdOx1-S recipients and led to expiration. Both cases were young (34 and 35 years) with no similar medical history (Table 4). Both incidence occurred post-first injection one after 2 and the other after 8 days [52, 53].

## Discussion

Massive vaccination campaigns have been launched since December 2020, applying mRNA vaccines and also the viral vector-based vaccine as well as inactivated viral-based and recombinant protein vaccines. By the end of January 2023, more than 5 billion individuals were fully vaccinated [42]. Thus, there is an increasing rate of reports over the adverse events associated with the administrated vaccines in the real world. General symptoms which have been normally screened include weakness, fever/chills, body pain, headache and local injection-site reactions. These symptoms are usually transient and do not normally need to be treated with specific medical care.

Herein, we discussed 64 cases of 40 studies who experienced unsolicited events after vaccinating against COVID-19. The applied vaccines included viral-vector and inactivated virus-based vaccines. We tried to select the case reports with new onset of the symptoms in whom the pre-existing comorbidity was not as same as the triggered adverse events. Various disorders were captured induced by different vaccines suggesting that the type of a specific regimen is not the only factor in outcomes. Moreover, there is not enough clues to support the triggered manifestations and their association with the applied vaccine. However, the healthy individuals who did not have any remarkable medical history and experienced serious events suggest that this potency of COVID-19 vaccine must be considered.

The common reported adverse events of ChAdOx1-S vaccine were pain at the injection site, fever, lethargy, muscle pain and headache which were mostly screened after the first dose of the vaccine [60]. The investigation by Pasteur Institute of Iran on ChAdOx1-S vaccine showed that a higher incidence of symptoms including fatigue, chills and myalgia were seen among homologous ChAdOx1-S recipients rather than those of a heterologous ChAdOx1-S/PastoCovac Plus group [61]. Moreover, irritability, nausea, myalgia, and chills some hours after vaccination with AZD1222 were reported in Nepal [62]. In addition to common adverse events, severe disorders were captured as postural drop in blood pressure, abdominal cramps, syncope and urticarial [63]. In this review, we found that ChAdOx1-S mostly led to neurological incidences, including encephalopathy [20], acute vestibular neuritis [34] and Guillain–Barre syndrome [38]. Although the safe administration of vaccines is a crucial factor, many unusual events following ChAdOx1-S vaccine have been reported. The Concern about neurological abnormalities regarding COVID-19 vaccines firstly rose in 2020 when some cases of Guillain–Barré syndrome and transverse myelitis were screened post-Oxford/ChAdOx1-S vaccine [64, 65]. In a recent comprehensive study on COVID-19 vaccines-related AEs, the most common observed neurological disorder was also Guillain–Barre syndrome. However, no association between the vaccines and the syndrome has been confirmed yet [66].

On the other hand, ChAdOx1-S has been the only cause of liver disorder in forms of Fulminant hepatitis [53] and acute liver failure which led to death in both cases in the present studied cases [52]. The previous studies on liver injury after COVID-19 vaccination of different platforms showed that mRNA-based vaccines and the vector-based ones both contributed to the captured disorders among which Pfizer‐BioNTech vaccine led to a liver failure [67]. Liver injury following COVID-19 vaccination is also investigated in a systematic review on individuals who got to Moderna (mRNA–1273), Pfizer–BioNTech BNT162b2 mRNA or ChAdOx1 nCoV-19 vaccine. Nevertheless, in those cases, pre-existing comorbidities was common as 69.6%, such as liver disease. The mortality rate due to live disorders was reported 4.3% [68].

The other adenovirus-based vaccine, Sputnik V, was also previously reported with fever/chills, general discomfort, headache arthralgia, myalgia, asthenia, tenderness as the common side effects [69, 70]. Similar to ChAdOx1-S, this vaccine mostly led to neurologic manifestations as Bell’s Palsy and Facial Paresis [31, 35]. Previous review study found Pfizer and Moderna vaccines as the most common reported causes of Bell’s palsy; however, COVAXIN and Sputnik V also led to it as well [71].

According to conducted studies in China, inactivated viral-based vaccines led to adverse events including injection site pain, lethargy and muscle pain 15.6% after the first and 14.6% after the second dose among the healthcare workers. The most common is pain at the injection site, followed by fatigue, muscle pain, and headache [72, 73]. Furthermore, two serious events as multiple sclerosis and emesis were also recorded with hospitalization requirement [74, 75].

In the present review, BBIBP–CorV vaccine resulted in corneal graft rejection in to cases a week after the first dose of injection [59]. In a study by Shah AP et al., four cases with a history of keratoplasty developed rejection after being vaccinated with mRNA-1273 [76]. This incidence has also been reported after adenovirus vector (AZD1222) and mRNA (BNT162) vaccines [77]. A systematic review also showed that Cornea rejection was the most reported organ rejection after vaccination against COVID-19, followed by kidney and liver rejections [78].

Dermal abnormalities have been the most frequent reported incidences after BBIBP–CorV vaccine among which new-onset lichen planus (LP) was observed in 6 cases [29]. Nevertheless, rare conditions were also screened, such as Toxic Epidermal Necrolysis [27], Morphea [25] and Pemphigus vulgaris [28]. Notably, of 28 skin disorders in the reported cases in this review, 20 cases got BBIBP–CorV vaccine. The other study from Iran evaluated the cutaneous reactions post-COVID‐19 vaccination which presented that most of the individuals showed symptoms after injection of ChAdOx1-S, BBIBP–CorV, Sputnik V, and COVAXIN vaccines [79].

Herpes zoster has been reported in case series and has also been documented in the Center of Disease Control following COVID-19 vaccines (VAERS). There are more than 1000 cases with mRNA vaccine-triggered herpes zoster in VAERS, mostly aged over 60 [80]. We also found a reported case of Herpes Zoster in a 60-year-old healthy woman 6 days after the first dose of BBIBP–CorV vaccine [25]. It has been suggested that molecular mimicry between the human components and vaccine‐induced proteins could lead to pathological autoantibodies generation and hence, autoimmunity accordingly [81].

As previously discussed, a quarter of the investigated cases experienced neurological involvements mostly as Guillain–Barré syndrome [38] and Bell's palsy caused [35] which were triggered by adenovirus-based vaccines and also BBIBP–CorV. Although the most incidences were captured post-first dose, a 60-year-old man presented Guillain–Barré syndrome 20 days after the BBIBP–CorV booster shot [82]. The correlation between Bell's palsy and vaccinations has been introduced previously, such as influenza H1N1 monovalent vaccine and intranasal inactivated influenza vaccine [83, 84]. Similar to other unknown mechanisms of vaccine induced problems, precise pattern of neurologic disorders is still under question. Some hypothetical thoughts though propose that autoimmune phenomenon as a result of host molecules mimicry with the vaccine antigen could activate auto‐reactive T cells [85].

Blood/vessels involvement were also reported in 6 cases as vasculitis, thrombotic thrombocytopenia, Cerebral venous sinus thrombosis, acquired thrombotic and lymphocytic vasculopathy caused by BBIBP–CorV and ChAdOx1-S [20, 44–46, 86]. Notably, all the cases presented the manifestation after the first dose of vaccination. A review study showed that thrombotic complications occurred 5–25 day post-first dose of ChAdOx1-S vaccinated individuals in which the thrombosis site was mostly in cerebral veins [87]. Although the exact mechanism of the events is not well-understood, the pre-existing antibodies such as heparin-PF4 antibody in the cases might give rise to the manifestations [88]. In addition, vasculitis precipitation has been also detected after other vaccines against hepatitis B virus (HBV), influenza virus and human papillomavirus (HPV) [89].

Although the discussed disorders have been screened post-vaccination, it is suggested that host immune responses are strongly the potential cause of the events. It is to say that, anti-spike immune responses might be linked to post-vaccine syndromes as all the vaccines against COVID-19 encode the whole or a part of spike protein. In addition to spike protein, anti-idiotypic antibodies can bind to the ACE-2 receptor as well [90]. Furthermore, the generated autoantibody stemming from molecular mimicry and independent immune-dysregulation may both contribute to a symptom onset [91]. However, it must be taken to attention that these mechanisms are still theoretical and have not been established as causal factors yet. Further studies are crucial to provide enough evidence.

In the present review, a comprehensive overview of COVID-19 vaccine-related case reports has been conducted. The classification of the vaccine-related AEs could make the recognition much easier and would contribute to further vaccine administration as well. Nevertheless, this study is only based on case reports which normally include inherent data quality and causality limitations and provides conclusive evidence of a causal relationship between the administered vaccines and the adverse events. Eventually, a comprehensive causality assessment in future studies to establish a more robust link between vaccinations and adverse events is of a high value owing to the fact that assessment the causality based on case reports would not be sufficient to draw general conclusions about vaccine safety. Eventually, case reports are often subject to selective reporting, which might have influenced the findings.

## Conclusion

The present review showed that various unsolicited adverse events have been captured as case reports in Iran. Interestingly, all the vaccine platforms could result in similar unsolicited events. Although, clinical trials provide safety data, the long-term evaluation of newly launched vaccines are essential to keep the public trust balanced.

COVID-19 has been the most recent mass vaccination program due to the broad range of infection world-wide. Thus, it is not far from view to face some rare disorders or late onset of a disease. Considering the advantage of the vaccination against SARS-CoV-2 which eventually led to the chaos management globally, the number of unsolicited AEs are not significant. However, the collective data from different populations would result in a better perspective for further vaccination program. The high risk individuals including those with a history of serious disease or comorbidities and those with immunodeficiency conditions should be vaccinated with the utmost caution. Future research to establish causality, the importance of continuous vaccine safety monitoring and the potential benefit–risk assessment for different populations are strongly recommended.

## Data Availability

Not applicable. A preprint has been previously published [92].
